# Openness in the NHS: a secondary longitudinal analysis of national staff and patient surveys

**DOI:** 10.1186/s12913-020-05743-z

**Published:** 2020-09-25

**Authors:** Imelda McCarthy, Jeremy Dawson, Graham Martin

**Affiliations:** 1 Aston Business School, Aston University, Birmingham, B4 7ET UK; 2 Management School, University of Sheffield, Conduit Road, Sheffield, S10 1FL England; 3 THIS Institute, University of Cambridge, Clifford Allbutt Building, Cambridge Biomedical Campus, Cambridge, CB2 0AH England

**Keywords:** Delivery of health care, Patient safety, Patient satisfaction, Quality of health care, Longitudinal studies

## Abstract

**Background:**

Improving openness—including candour when things go wrong, and willingness to learn from mistakes—is increasingly seen as a priority in many healthcare systems. This study explores perceptions of openness in England before and after the publication of the Francis report (2013), which examined failings of openness at one English hospital. We examine whether staff and patients’ views on openness, and experiences of giving voice to concerns, have changed since the report’s publication for better or worse.

**Methods:**

Organisational-level data was collated for all trusts from the NHS National Staff Survey (2007–2017), NHS Acute Inpatient Survey (2004–2016) and NHS Community Mental Health Service User Survey (2007–2017). Survey items related to openness were identified and longitudinal statistical analysis conducted (piecewise growth curve and interrupted latent growth curve analysis) to determine whether there was evidence of a shift in the rate or direction of change following publication of the Francis report.

**Results:**

For some variables there was a discernible change in trajectory after the publication of the Francis report. Staff survey variables continued to rise after 2013, with a statistically significant increase in rate for “fairness and effectiveness of incident reporting procedures” (from + 0.02 to + 0.06 per year; *p* < .001). For the patient surveys, the picture was more mixed: patient views about information provided by accident and emergency staff rose from a 0.3% increase per year before 2013 to 0.8% per year afterwards (*p* < .01), and inpatients being involved in decision making increased from a 0.4% rise per year before 2013 to 0.8% per year afterwards (*p* < .01); however, there were not rises in the other questions. Mental health patients reported a decrease after 2013 in being listened to (decreasing at a rate of 1.9% per year, *p* < .001).

**Conclusions:**

Data suggest that the Francis inquiry may have had a positive impact on staff and acute inpatients’ perceptions and experiences of openness in the NHS. However such improvements have not transpired in mental health. How best to create an environment in which patients can discuss their care and raise concerns openly in mental health settings may require further consideration.

## Background

Calls have been made for greater openness within the National Health Service (NHS) in England, with the intention of creating of a culture ‘*where mistakes are acknowledged and learned from’* [1], thus attempting to counteract the effects of past failings that have come to public attention in recent years [2, 3]. Elsewhere in the world, policy makers have similarly identified issues with openness about the quality of care, ranging from day-to-day shortcomings in reviewing and learning from incidents [4], to major scandals involving persistent failures and efforts to conceal them [5]. Greater openness and transparency about such issues, among healthcare staff and between staff and their patients, is often suggested as a means of addressing these issues and improving the quality of care [6]. In England, a notable case with important consequences for policy is that of Stafford Hospital, where concerns about poor care and high patient mortality rates came to light in the late 2000s. Both the problems themselves and the fact that they continued for so long unchecked were the focus of extensive media coverage, reflection within the NHS and government attention. Sir Robert Francis chaired two inquiries [2, 7] which cited a lack of vigilance on the part of hospital administrators and system regulators as contributing factors to the tragedies that occurred. Disconcertingly, it was recognised that many employees were aware of the problems before they became public but were either reluctant to speak up [8] or had their concerns disregarded by those in power [9].

The events at Stafford Hospital were tragic and extreme but may not have been unique. Accordingly the Francis inquiry [2] called for cultural change across the whole NHS, in terms of greater openness cascading from the top to the bottom of all NHS trusts (provider organisations within the NHS), to prevent mistakes and promote learning. It demanded a system that was more open in providing and using information about performance, more transparent in the way it made such information available to staff and patients, and more candid with patients when things go wrong in the course of their care [2]. The Department of Health acted on these recommendations by introducing the Duty of Candour [10], changes to the reporting of Care Quality Commission (CQC) inspections [3], changes to the way serious incidents are investigated [11], and introducing Freedom to Speak up Guardians in all NHS trusts, among other measures [12]. Similar initiatives have been introduced elsewhere in the world—for example communication-and-resolution programmes in New Zealand and the United States, and worldwide efforts to improve the quality and impact of learning from incidents. However, past work suggests that such efforts may be more easily accomplished in acute healthcare than in some other settings, for example mental health. Such changes were intended to create an NHS culture of openness and honesty—two factors key to organisational trust [13]. Research suggests that trust has a beneficial impact on working life, including increased job satisfaction and organisational effectiveness [14]. Trust is also important to patients and has been associated with positive perceptions of the quality of care they receive [14].

The impact these initiatives remains unclear; accordingly a longitudinal research design was applied using data from NHS annual surveys of staff and patients to explore perceptions of openness since the publication of the Francis report (2013) to answer the research question: Are staff and patients’ views on openness and experiences of giving voice to concerns changing through time, for better or worse?

## Methods

This study used a longitudinal, observational design, examining routinely collected annual data aggregated to the organisational level. Full details for all surveys and years can be found in Table 1. Data were analysed from the NHS National Staff Survey (hereafter ‘Staff Survey’) years 2007–2017. The Staff Survey collects staff views about working in their NHS trust [15].

**Table 1:** Response rates for NHS Staff and Patient Surveys

Survey	Year	Number of questionnaires sent out*	Number of questionnaires returned	Response rate	Number of Trusts
**NHS National Staff Survey**	2007	291,843	157,667	54%	392
	2008	289,919	159,691	55%	360
	2009	289,277	157,450	54%	387
	2010	311,098	167,736	54%	390
	2011	250,000	134,967	54%	365
	2012	203,188	101,169	50%	259
	2013	416,313	203,028	49%	264
	2014	603,937	255,150	42%	289
	2015	722,811	298,817	41%	296
	2016	948,640	414,330	44%	316
	2017	1,067,266	478,872	45%	309
**NHS Acute Inpatient Survey**	2004	142,432	88,308	62%	169
	2005	136,937	80,793	59%	164
	2006	136,769	80,694	59%	166
	2007	135,623	75,949	56%	165
	2008	134,415	72,584	54%	165
	2009	133,362	69,348	52%	161
	2010	132,696	66,348	50%	161
	2011	133,704	70,863	53%	161
	2012	126,480	64,505	51%	156
	2013	127,435	62,443	49%	156
	2014	125,709	59,083	47%	154
	2015	176,843	83,116	47%	149
	2016	176,932	77,850	44%	149
**NHS Community Mental Health Service User Survey**	2007	41,842	15,900	38%	69
	2008	41,014	14,355	35%	68
	2009	n/a	n/a	n/a	n/a
	2010	53,746	17,199	32%	66
	2011	52,852	17,441	33%	65
	2012	49,619	15,878	32%	61
	2013	46,552	13,655	29%	57
	2014	46,552	13,500	29%	57
	2015	41,650	11,695	29%	52
	2016	49,300	13,254	28%	58
	2017	47,600	12,139	26%	58

The NHS Acute Inpatient Survey (‘Inpatient Survey’) is conducted each year within acute care. The survey collects patients’ views about their stay in hospital [16].

Data from the NHS Community Mental Health Service User Survey (‘Mental Health Survey’) was sourced for years for 2007–2017 (excluding 2009 as no survey was conducted that year). The survey collects patients’ views about the care they received whilst using mental health services [17].

The Francis report called for improvements in “openness, transparency and candour,” defined respectively as

- “the proactive provision of information about performance, negative as well as positive” (openness),
- “the provision of facilities for all interested persons and organisations to see the information they need properly to meet their own legitimate needs in assessing the performance of a provider in the provision of services” (transparency), and
- “the volunteering of all relevant information to persons who have, or may have, been harmed by the provision of services, whether or not the information has been requested” (candour) [2].

We sought to operationalise these values by choosing items from the three surveys that related most closely to them. A list of questions from each survey that had remained consistent over a minimum of 6 years, including the period from 2011 to 2014, was compiled. These were then examined individually by five members of the research team (including the three authors of this article, one medical sociologist, and one other health services researcher). Each was assessed for whether it was strongly related, moderately related, or not strongly related to any of the three definitions of openness, transparency and candour above. Where questions were identified by all as being strongly related, they were chosen for the analysis. Where they were identified by some as being strongly related, but by others as being only moderately related, these were discussed by the research team and agreement reached about whether they should be included. All members of the research team approved the list of items before analysis began. Analysis included all trusts that had remained single organisations over the period. The final set of variables included is shown in Table 2, which also describes how each score was calculated from the original questions. The scoring mechanisms used in routine analysis and publication of the surveys was retained, as these are established, validated measures.

**Table 2:** Details of questions used in study

Survey	Variable	Original question & scoring	Aggregation method
**NHS Staff Survey**	Good communication between managers and staff	Four questions with 5-point Likert-scale responses (ranging from “Strongly disagree” to “Strongly agree”): • Senior managers here try to involve staff in important decisions. • Communication between senior management and staff is effective. • I know who the senior managers are here. • Senior managers act on staff feedback.	% employees who agreed or strongly agreed with at least three of the four statements
	Can contribute towards improvements	Three questions with 5-point Likert-scale responses (ranging from “Strongly disagree” to “Strongly agree”): • There are frequent opportunities for me to show initiative in my role. • I am able to make suggestions to improve the work of my team / department. • I am able to make improvements happen in my area of work.	% employees who agreed or strongly agreed with at least two of the three statements
	Fairness and effectiveness of incident reporting procedures	Seven questions with 5-point Likert-scale responses (ranging from “Strongly disagree” to “Strongly agree”): • My organisation treats staff who are involved in an error, near miss or incident fairly. • My organisation encourages us to report errors, near misses or incidents. • My organisation treats reports of errors, near misses or incidents confidentially. • My organisation blames or punishes people who are involved in errors, near misses or incidents. • When errors, near misses or incidents are reported, my organisation takes action to ensure that they do not happen again. • We are informed about errors, near misses and incidents that happen in the organisation. • We are given feedback about changes made in response to reported errors, near misses and incidents.	Average scale score calculated for each individual (based on 1 = “Strongly disagree” to 5 = “Strongly agree”); these then averaged across all individuals in each organisation.
**NHS Acute Inpatient Survey**	Information about condition or treatment	A single question, with wording “While you were in the A&E Department, how much information about your condition or treatment was given to you?” Responses “The right amount” (scored as 100), “Too much” or “Too little” (scored as 50), or “I was not given any information” (scored as 0)	Scores averaged across all respondents for the organisation
	Involvement in decisions about care and treatment	A single question, with wording “Were you involved as much as you wanted to be in decisions about your care and treatment?” Responses “Yes, definitely” (scored 100), “Yes, to some extent” (scored 50), or “No” (scored 0)	Scores averaged across all respondents for the organisation
	Ability to talk about worries and fears	A single question, with wording “Did you find someone on the hospital staff to talk to about your worries and fears?” Responses “Yes, definitely” (scored 100), “Yes, to some extent” (scored 50), or “No” (scored 0) (A fourth response option, “I had no worries or fears”, was ignored for this calculation)	Scores averaged across all valid respondents for the organisation
**NHS Community Mental Health Service User Survey**	Listening carefully	A single question, with wording “Did the person or people you saw listen carefully to you?” (N.B. before 2010 this question specifically referenced “your psychiatrist”, rather than “people or person you saw”) Responses “Yes, definitely” (scored 100), “Yes, to some extent” (scored 50), or “No” (scored 0) (A fourth response option, “Don’t know/can’t remember”, was ignored for this calculation)	Scores averaged across all valid respondents for the organisation
	Enough time to discuss needs and treatment	A single question, with wording “Were you given enough time to discuss your needs and treatment?” Responses “Yes, definitely” (scored 100), “Yes, to some extent” (scored 50), or “No” (scored 0) (A fourth response option, “Don’t know/can’t remember”, was ignored for this calculation)	Scores averaged across all valid respondents for the organisation
	Formal meetings to review ongoing care	A single question, with wording “In the last 12 months have you had a formal meeting with someone from NHS mental health services to discuss how your care is working?” Responses “Yes” (scored 100) or “No” (scored 0) (A third response option, “Don’t know/can’t remember”, was ignored for this calculation)	Scores averaged across all respondents for the organisation
	Treatment with respect and dignity	A single question, with wording “Did you feel that you were treated with respect and dignity by NHS mental health services?” Responses “Yes, always” (scored 100), “Yes, sometimes” (scored 50), or “No” (scored 0)	Scores averaged across all respondents for the organisation

Longitudinal statistical analysis was conducted in Mplus Version 8. This modelled staff and patient survey outcomes over time to determine any change in responses to questions relating to openness. All analysis was conducted at the trust level, with individual responses aggregated to create the mean, or a percentage score, depending on the type of question. To search for the optimal growth trajectory, piecewise growth curve analysis [18] was conducted to compare the intercept (the starting level) and the slope (rate of change over the period of interest) either side of 2013 for each survey outcome. For illustration, a Piecewise Growth Curve Model (PGCM), with a breakpoint at 2013, for Staff Survey data available from 2007 to 2017, would have two linear trajectories, the initial piece representing data 2007–2013 and the latter piece representing data 2013–2017. The Wald test is then used to test whether the initial trajectory differs significantly from the latter trajectory (a larger Wald value representing a difference that is less likely to be due to chance). This model effectively tests whether there is a general trend over time that changed following the publication of the Francis report in 2013.

PGCM analysis assumes continuous change following a turning point - however this is not always the case. Change may be temporary before a trajectory returns to its original path, or takes a different direction. Accordingly we tested for the possibility of an interrupted time series using Interrupted Latent Growth Curve Model (ILGM) analysis [19]. Building upon the illustration above, an ILGM with an interrupt 2013–2014, would allow an immediate, separate change between 2013 and 2014, and would test (using the Wald test) for a significant difference between the mean of the initial and latter piece of the growth curve at the point of interruption. This model effectively tests whether the publication of the Francis report in 2013 was followed by an immediate change in experience, followed by a (possibly) different trend following this. All models allow for each trust to have its own trajectory for each piece (and step change where required); these are modelled as random effects so that an overall mean effect is calculated and tested. It is these mean effects that are reported here.

## Results

**Fig. 1:**
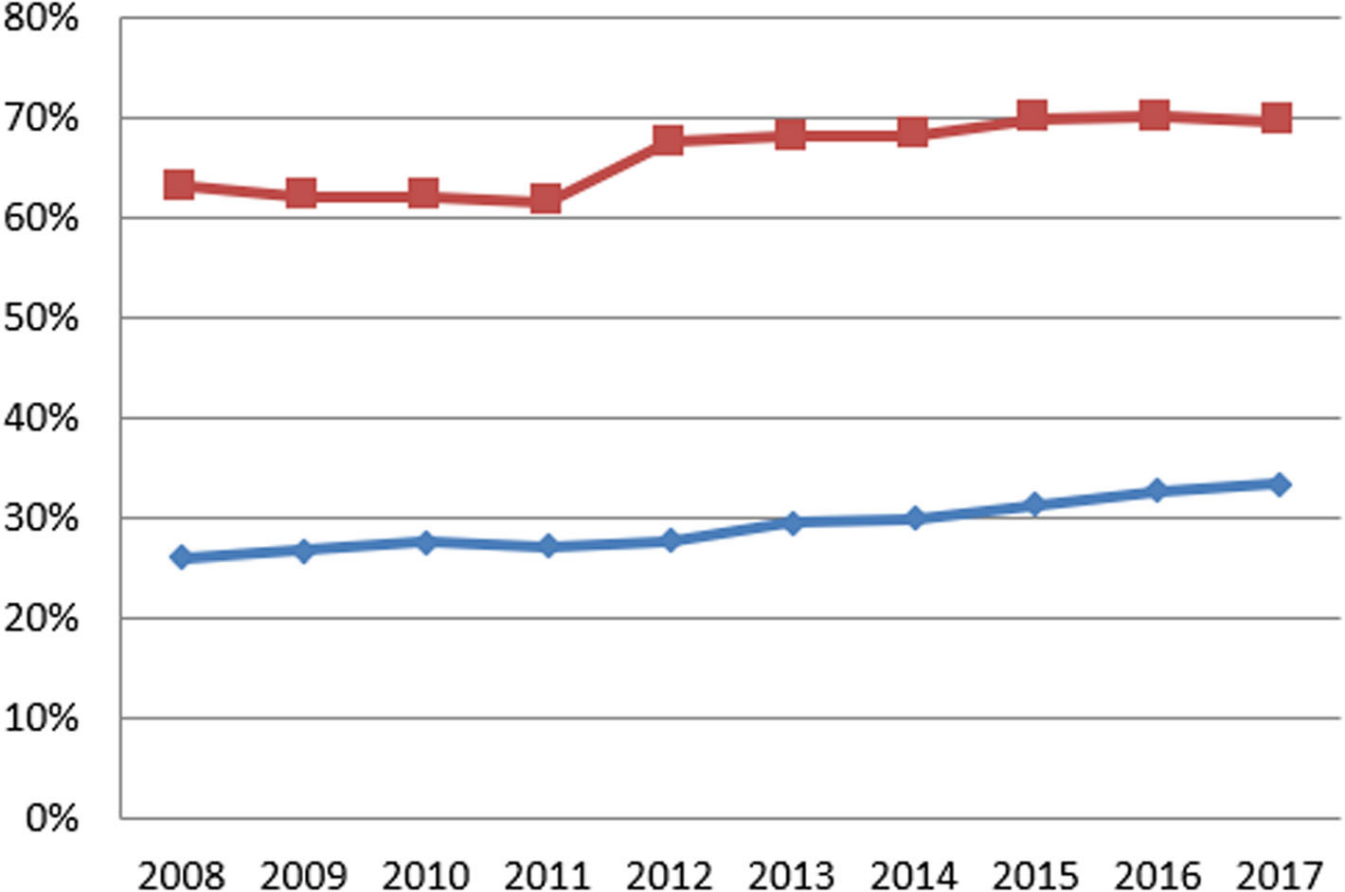
Annual average percent change for NHS National Staff Survey questions

**Fig. 2:**
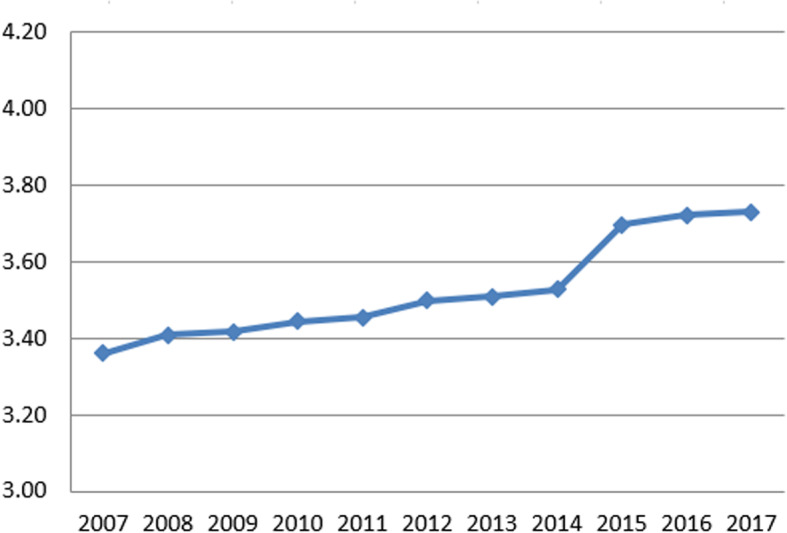
Annual average percent change for NHS National Staff Survey questions

**Fig. 3:**
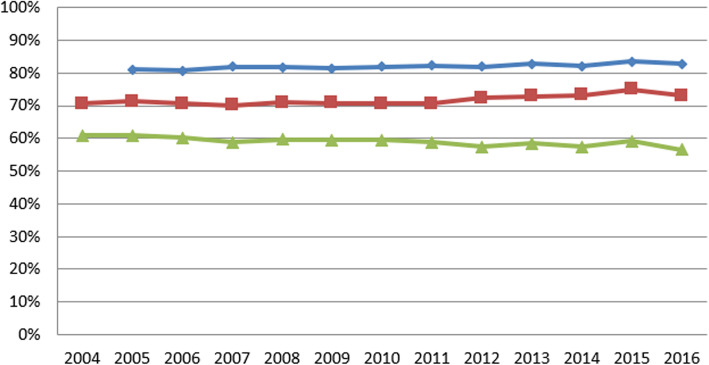
Annual average percent change for NHS National Staff Survey questions

**Fig. 4:**
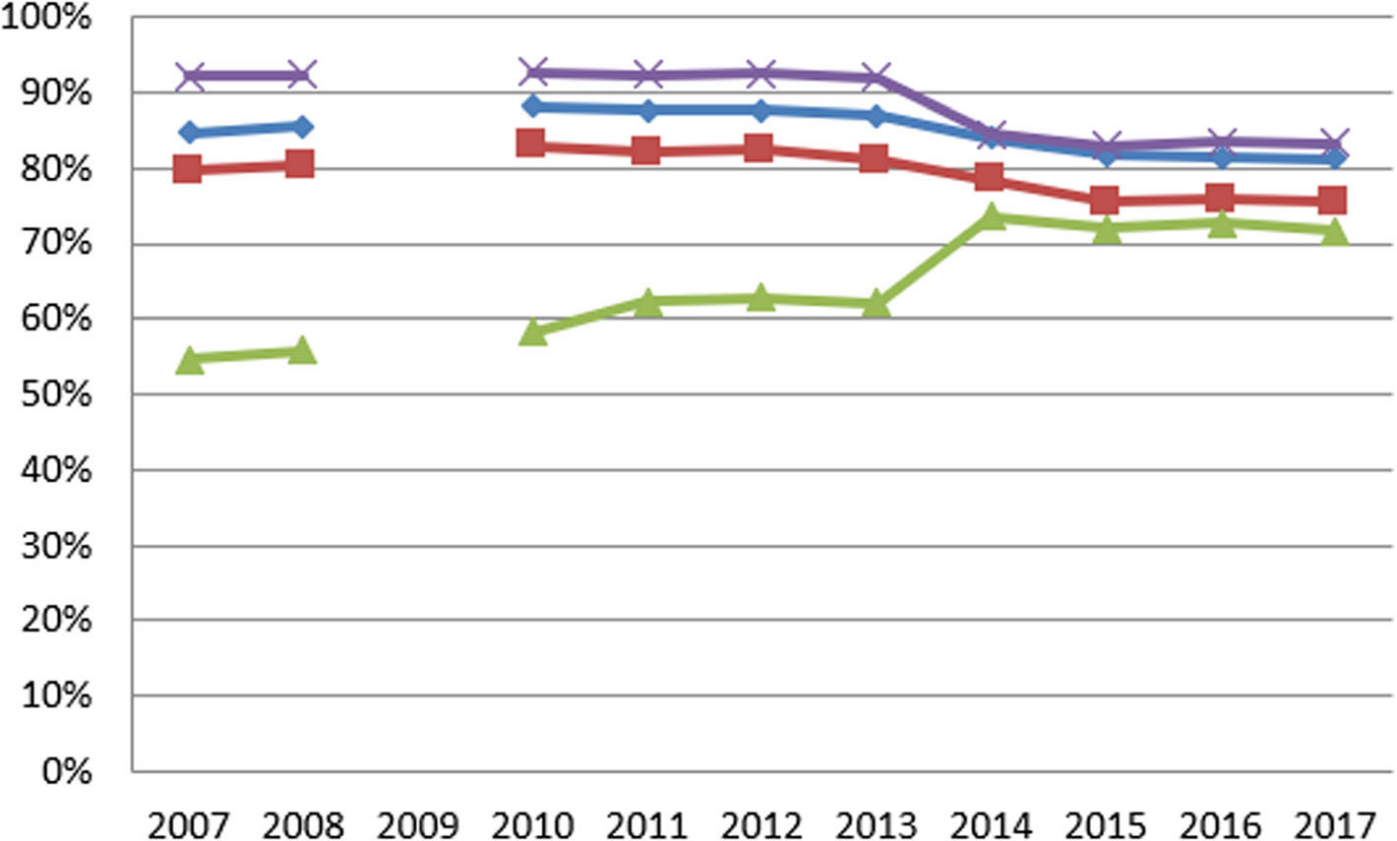
Annual average percent change for NHS National Staff Survey questions

Table 3 shows summary statistics for the ten variables we used. Summary statistics are shown for the first and final year only to give an indication of the level of spread and overall change; summary statistics for all variables for all years can be found in the supplementary material. The spread remained fairly consistent across years, although there were changes in the mean values across time for many variables, sometimes relatively gradually, and sometimes with big jumps from 1 year to the next: this is what was tested with our models, and these changes are shown in Figs. 1, 2, 3 and 4 (with summaries of the models shown in Table 4). The results presented here assume a general pattern of growth in the direction of the trajectory stated, unless specified otherwise. The models for the staff survey and inpatient survey had adequate or good levels of fit; the models for the mental health survey, however, had poor fit, indicating that these models do not explain the data as well as would be hoped. To allow comparison of these models with competing models, our supplementary material includes a table where the fit of these models for all variables is compared with that from three competing models; this indicates that, even when fit is poorer, it is still generally superior to these competing models.

**Table 3:** Summary statistics for variables in first and final year

Survey (Data year)	Variable (years measured)	First year			Final year		
		Mean (SD)	Median	Range	Mean (SD)	Median	Range
NHS Staff Survey	Good communication between managers and staff (2008–2017)	26% (7%)	26%	(4, 57%)	33% (6%)	34%	(14, 48%)
	Can contribute towards improvements (2008–2017)	63% (8%)	64%	(31, 81%)	70% (6%)	70%	(40, 79%)
	Fairness and effectiveness of incident reporting procedures (2007–2017)	3.36 (0.11)	3.36	(2.81, 3.67)	3.73 (0.12)	3.74	(3.17, 4.03)
NHS Acute Inpatient Survey	Information about condition or treatment (2005–2016)	81% (4%)	80%	(73, 93%)	83% (4%)	82%	(74, 96%)
	Involvement in decisions about care and treatment (2004–2016)	71% (5%)	70%	(59, 84%)	73% (5%)	72%	(63, 89%)
	Ability to talk about worries and fears (2004–2016)	61% (7%)	61%	(43, 81%)	56% (6%)	55%	(45, 77%)
NHS Community Mental Health Service User Survey	Listening carefully (2007–2017)	85% (2%)	85%	(80, 90%)	81% (3%)	82%	(70, 87%)
	Enough time to discuss needs and treatment (2007–2017)	80% (3%)	80%	(72, 85%)	80% (3%)	75%	(59, 82%)
	Formal meetings to review ongoing care (2007–2017)	55% (10%)	53%	(37, 75%)	72% (5%)	71%	(59, 83%)
	Treatment with respect and dignity (2007–2017)	92% (2%)	93%	(88, 96%)	83% (4%)	84%	(71, 88%)

(Full summary statistics for each year can be found in the supplementary material)

**Table 4:** Piecewise Growth Curve Model (PGCM^a^) and Interrupted Latent Growth Curve Model (ILGM^b^) Slope Mean Differences

Survey	Variable	Model	Wald Test	CFI	RMSEA	Initial Slope Mean	Interrupt Slope Mean	Latter Slope Mean
**NHS National Staff Survey**	Good communication between managers and staff (2008–2017)	PGCM	2.94	.965	.063	0.2%		0.7%
		ILGM	7.70**	.969	.061	0.2%	−0.1%	0.8%
	Can contribute towards improvements (2008–2017)	PGCM	0.11	.869	.149	1.0%		0.7%
		ILGM	4.14*	.875	.147	1.0%	0.0%	0.7%
	Fairness and effectiveness of incident reporting procedures (2007–2017)	PGCM	13.64***	.831	.161	0.02		0.06
		ILGM	8.45**	.832	.161	0.02	0.00	0.06
**NHS Acute Inpatient Survey**	Information about condition or treatment (2005–2016)	PGCM	0.05	.865	.087	0.3%		0.6%
		ILGM	14.37**	.885	.080	0.3%	−0.1%	0.8%
	Involvement in decisions about care and treatment (2004–2016)	PGCM	9.03**	.901	.114	0.4%		0.8%
		ILGM	0.00	.900	.114	0.4%	0.0%	0.8%
	Ability to talk about worries and fears (2004–2016)	PGCM	4.27*	.952	.077	−0.0%		0.2%
		ILGM	0.08	.952	.078	−0.0%	− 0.0%	0.2%
**NHS Community Mental Health Service User Survey**	Listening carefully (2007–2017)	PGCM	11.83***	.207	.175	0.3%		−1.9%
		ILGM	3.03	.226	.174	0.3%	0.5%	−1.8%
	Enough time to discuss needs and treatment (2007–2017)	PGCM	8.30**	.363	.154	0.3%		−1.8%
		ILGM	0.47	.360	.155	0.3%	−0.2%	−1.7%
	Formal meetings to review ongoing care (2007–2017)	PGCM	5.16 *	.033	.198	1.9%		1.0%
		ILGM	5.85**	.098	.192	1.8%	3.2%	0.5%
	Treatment with respect and dignity (2007–2017)	PGCM	6.04**	.000	.243	−0.4%		−2.5%
		ILGM	17.54***	.118	.206	−0.1%	−6.6%	−0.4%

*p < 0.05; **p < 0.01; ***p < 0.001

^a^ PGCM has breakpoint at 2013

^b^ ILGM has step change at 2013–2014

(Fit statistics for competing models can be found in the supplementary material)

### Staff survey

Good communication between managers and staff increased from 2008 to 2017 (national averages ranging from 26.0% to a maximum of 33.5%). According to the IGLM (which had better fit), between 2008 and 2013 this increase represented growth of 0.2% per annum (reflecting an average trust-level change of 0.2% in this score each year). There was a slight decrease between 2013 and 2014 (significant at *p* < 0.01) of − 0.1%. Rates of growth returned to a positive trajectory of 0.8% per annum between 2013 and 2017: a non-significant (*p* > 0.1) increase compared to previous years.

Opportunities for staff to contribute towards improvements at work increased between 2008 and 2017 (ranging from 61.6 to 70.2%). According to the IGLM (which had slightly better fit), an initial increase of 1.0% per annum between 2008 and 2013 was followed at slower rate between 2013 and 2017 of 0.7% per year. However there was a period of stagnation between 2013 and 2014 (*p* < 0.05).

There was an overall increase in perceptions of the fairness and effectiveness of incident reporting procedures between 2007 and 2017 (ranging from 3.36 to maximum of 3.73 on a 1–5 Likert scale). There was little to choose between the two models: both showed that between 2007 and 2013 this increase averaged 0.02 scale points per year and continued at a faster rate between 2013 and 2017 with an average annual increase of 0.06 scale points. The difference between rate of change pre- and post-2013 was significant (*p* < 0.001). (Note that the Y-axis in Fig. 2 ranges from 3.0 to 4.2 – this is because this encapsulates the whole range of trust-level values observed on this variable.)

### Inpatient survey

Between 2005 and 2016 there was an improvement in patients’ views about the amount of information provided by A&E staff (ranging from 80.8 to 83.6%). According to the better-fitting IGLM, between 2005 and 2013 this increase averaged 0.3% per year. Between 2013 and 2014 there was a slight decrease of − 0.1% (*p* < 0.01), before returning to a positive trajectory between 2013 and 2016 of 0.8% per year – a significant increase (*p <* 0.01) compared with the pre-2013 trajectory.

From 2004 to 2016 positive responses about involvement in decisions about your care and treatment ranged from 70.3 to 75.1%. The models fitted equally well, and both indicated that between 2004 and 2013 these increased by an average of 0.4% per year. This continued at a slightly faster rate between 2013 and 2016, at an average of 0.8% per year. The difference in rates of change pre and post 2013 was significant at *p <* 0.01.

Overall between 2004 and 2016 scores for the whether patients had access to someone on the hospital staff whom they could talk to about their worries and fears ranged from 56.6 to 61.5%. According to the slightly better-fitting PGCM, between 2004 and 2013 scores decreased by an average of 0.04% per year; however between 2013 and 2016, this trend changed to one of annual fluctuations in either direction, and on average over this period a slight increase (*p* < 0.05) of 0.2% per year.

### Mental health survey

For the question, ‘Did the person or people you saw listen carefully to you?’ scores ranged from 81.5 to 88.3%. According to the PGCM (which had slightly better fit), from 2007 to 2013 this increased by an average of 0.3% per year; after this (until 2017) there was a significant decrease (*p* < 0.001) at an average of − 1.9% per year, suggesting patients may not feel as listened to as they once did. It must be noted that the wording of this question has altered slightly: before 2010, participants were asked specifically about their psychiatrist, but in latter years, the question referred to their experience of the service generally.

Scores for the question ‘Were you given enough time to discuss your needs and treatment?’ ranged from 75.5 to 83.1%. According to the PGCM (which had slightly better fit), there was an increase from 2007 to 2013 by an average of 0.3% per year; after this (and up until 2017) there was a significant decrease (*p* < 0.01) at an average of − 1.8% per year, indicating patients are becoming less satisfied with the amount of consultation time available to them. Again, there is a change in the phrasing of this question from 2010 as above.

When asked whether they had had a formal meeting with someone in the previous 12 months to discuss how their care is working, responses ranged from 55.1 to 73.9%. Both models fitted poorly, but the ILGM was slightly better. Positive responses to this question increased between 2007 and 2013 by an average of 1.8% per year. There was a significant increase (*p* < 0.01) at a rapid rate from 2013 to 2014 by 3.2%. From 2013 to 2017 there was still an increase but at a slower rate on average of 0.5% per year – a significant deceleration (*p* < 0.001) compared to previous years.

Scores for the question, ‘In the last 12 months, did you feel that you were treated with respect and dignity by NHS mental health services?’ ranged from 83.0 to 92.8%. The IGLM was slightly better fitting, and according to this, between 2007 and 2013 this variable decreased by an average of − 0.1% per year; this continued but at a faster rate of − 6.6% (*p <* 0.001) between 2013 and 2014. Between 2013 and 2017 there was still a decrease but at a slower rate of − 0.4% per year – a slight but non-significant (*p* > 0.1) worsening compared to the 2007 to 2013 timeframe. All of these findings for the mental health survey should be taken alongside the knowledge that the fit of these models is poor; while the patterns described here are still correct on average, there will have been many other unmeasured factors that were causing changes in the scores and resulting in poorer fit. Importantly, fit was not improved by altering the year in which there was a change in the growth curve or step change.

## Discussion

A discernible change was observed amongst Staff, Inpatient and Mental Health Survey data in the rate and sometimes the direction of change after the publication of the Francis report in 2013.

For Staff Survey variables relating to openness there were some significant improvements after the publication of the Francis report. This included an increased upwards trajectory in the fairness and effectiveness of incident reporting procedures (which was already improving before the Francis report). For communication between managers and staff, and opportunities for staff to contribute towards improvements at work, the increases continued after publication of the Francis report, although not at a higher rate than before.

For Inpatient and Mental Health Survey measures the picture was more mixed. The general trend for the Inpatient Survey was generally positive, with increases at a faster rate during the second period. Specifically, from 2013 satisfaction with the amount of information given to patients in A&E about their condition or treatment increased at a faster rate, and patients’ satisfaction with their involvement in decisions about their care and treatment also increased more sharply.

For the Mental Health Survey the pattern of change was rather different. Patients continued to report better access (as indicated by whether they had attended a meeting to discuss their care in the last year), though at a slower rate after 2014. However, levels of satisfaction indicated in other questions relating to openness deteriorated: patients felt less listened to, believed they were not given enough time to discuss their care, and felt treated with less respect and dignity compared to previous years. Such findings are perhaps noteworthy in view of recent commentary on the disparity between physical and mental health, which includes an imbalance between perceptions, services, resources and funding in favour of physical health [20], as well as indications that realising the values of openness and transparency may be more challenging in mental health and community settings than in acute healthcare organisations [21].

Mental health has long been considered the poor relation of the NHS [22], and in much of the world, patients with mental health conditions suffer stigma, and mental healthcare is underfunded compared to physical health [23]. Only a properly resourced mental health service can assure decent and humane outcomes for patients and their families [24]. When services are not properly resourced change is often slow or limited [25]. Our findings here suggest that increased policy attention to the importance of mental health has not yet translated into improved patient experiences, at least in relation to matters relating to openness; indeed, over the period since the publication of the Francis report, the disparity has increased. They also need to be interpreted bearing in mind that the fit of these models for the mental health survey was poor: although the patterns described by the models do give a helpful picture in indicating how things have changed over the years, it is likely that if other types of model were fitted, particularly those that took into account other external factors affecting mental health services over this period, they would result in better fit and more helpful models.

### Limitations

The paper is not able to explore causal effects between the Francis inquiry and openness because there was no control group. The breakpoint chosen was the year of the publication of the second Francis inquiry, suggesting the inquiry may have had an impact, although of course action in response to the issues at Stafford is likely to have been more diffuse, with organisations making changes in anticipation of the inquiry’s findings, and continuing to act as policies were introduced over the years after the inquiry’s publication. However, we cannot evidence a causal relationship, since other major changes in the NHS were also taking place at the time – most notably the Health and Social Care Act [26] that came into effect on the 1 April 2013 – which may have had an equal or greater impact. The response rates of the three surveys included are mixed, ranging from 26 to 62%. For all three, there is a general downward trend in response rate through time, which may have an impact on responses that is difficult to predict. There are many possible reasons for this, including increased survey fatigue, higher workload (in the case of the staff survey), and a lowering of survey profile over time. Dissatisfaction with the service may be a factor also, for the patient surveys at least, although there is little other evidence that this is the case. In addition, just as we cannot know whether changes made by the NHS in response to the Francis report directly resulted in our findings, there is also a chance that the findings of the Francis report will have influenced staff and patients in the way they answered survey questions.

Additionally, it may appear that some of the changes found were small in numerical terms. It is difficult to pinpoint exactly what is a clinically or socially relevant change, but it is worth noting that even small changes can produce an important overall population difference when it is multiplied across a service that includes hundreds of organisations, hundreds of thousands of staff, and millions of patients.

Finally, we were unable to take into account different actions that trusts will have acted differently according to their own responses to issues as they occurred, and there is a great deal of variety between types of trust, even within the same broad type (e.g. large teaching hospitals compared with small district general hospitals).

## Conclusions

Our findings suggest that from the perspective of staff and to some extent inpatients in acute hospitals, experiences of aspects of care relating to openness have improved through time. While this cannot be attributed directly to the Francis inquiries or to the policy interventions that have followed, our data do suggest that in aggregate, and along with other influences on healthcare provision in England, efforts to improve openness in the sector are having a positive impact. There is evidence that similar initiatives elsewhere in the world, for example efforts to encourage disclosure and reconciliation following serious incidents, have had a positive impact on the views of openness of staff and patients, although ensuring that such policies are implemented in a sensitive and patient-centred way is crucial [27–29].

The UK Government has pledged £2.3bn in funding to improve mental health services as part of a ten-year plan focused on prevention and early detection [30]. This effort reflects a longstanding policy commitment to ‘parity of esteem’ between physical and mental health services in the NHS. Ensuring that patients feel able to discuss their conditions and raise concerns about care is an important component of ensuring high-quality care, but our findings suggest a worrying and sustained trend for several indicators as assessed by mental health service users. Our findings point towards the scale of the challenge facing ambitions to improve quality of care in mental health, as many indicators of an open culture, as perceived by mental health patients, deteriorate or stagnate, while their counterparts in the acute inpatient survey improve over the same period. Policymakers might consider how they can support a culture of openness in the mental health sector of the NHS, noting that many of the interventions introduced after the publication of the Francis inquiry were modelled on the acute hospital setting, and do not translate so easily into settings where care may be dispersed across sites or provided primarily in the community [21]. Alternative approaches, designed around the particularities of mental healthcare provision and the needs of mental health service users, may be required to optimise opportunities for voice in this setting.

## Supplementary information

**Additional file 1.**

## Data Availability

The datasets used in this study are derived from publicly available data from national NHS surveys. The datasets analysed are available from the corresponding author on reasonable request.

## Abbreviations

A&E: Accident and Emergency
CQC: Care Quality Commission
NHS: National Health Service
PGCM: Piecewise Growth Curve Model
