# Correction to: Openness in the NHS: a secondary longitudinal analysis of national staff and patient surveys

**DOI:** 10.1186/s12913-020-05832-z

**Published:** 2020-10-30

**Authors:** Imelda McCarthy, Jeremy Dawson, Graham Martin

**Affiliations:** 1 Aston Business School, Aston University, Birmingham, B4 7ET UK; 2 Management School, University of Sheffield, Conduit Road, Sheffield, S10 1FL England; 3 THIS Institute, University of Cambridge, Clifford Allbutt Building, Cambridge Biomedical Campus, Cambridge, CB2 0AH England

**Correction to: BMC Health Serv Res (2020) 20:900**

**https://doi.org/10.1186/s12913-020-05743-z**

Following the publication of the original article [1], it was noted that due to a typesetting error Figs. 1, 2, 3 and 4 have not included the figure legends.

**Fig. 1:**
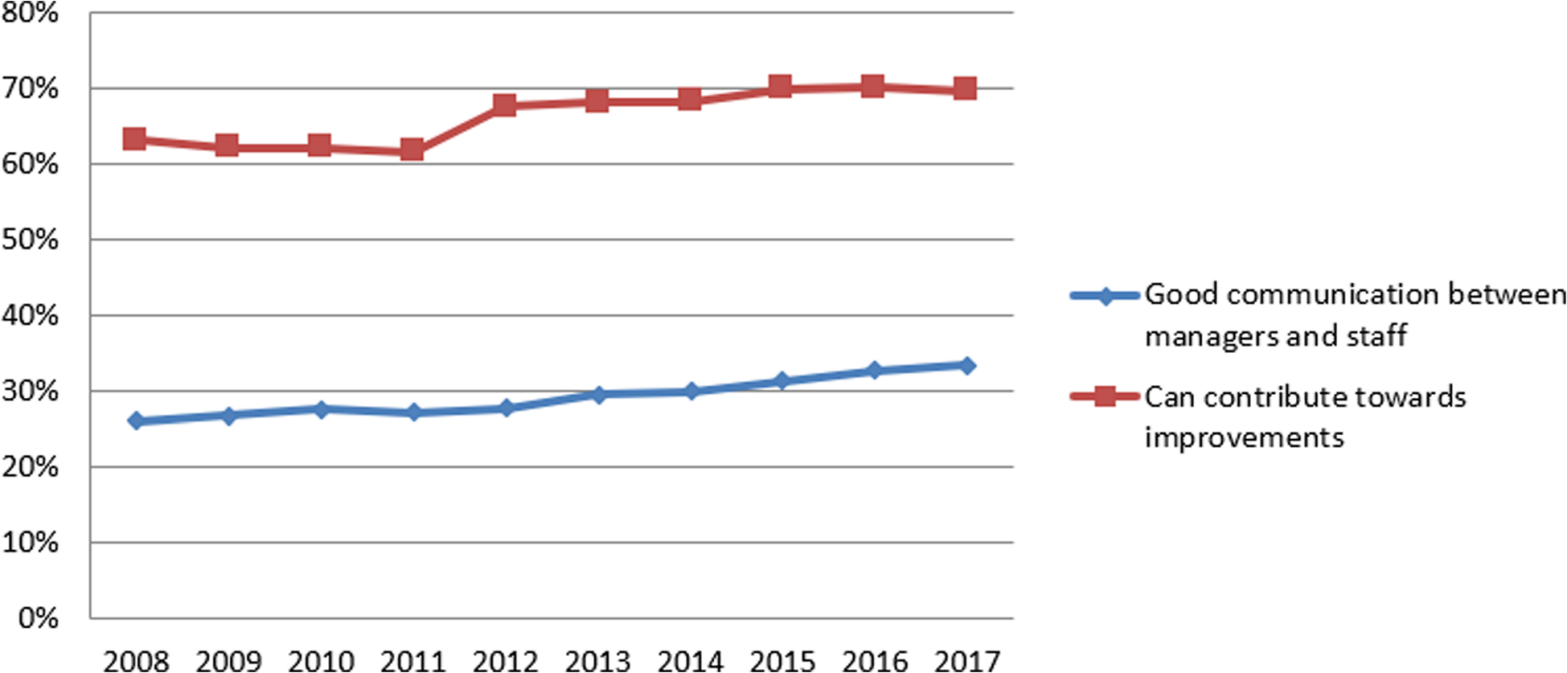
Annual average scores for NHS Staff Survey questions (percentage scores)

**Fig. 2:**
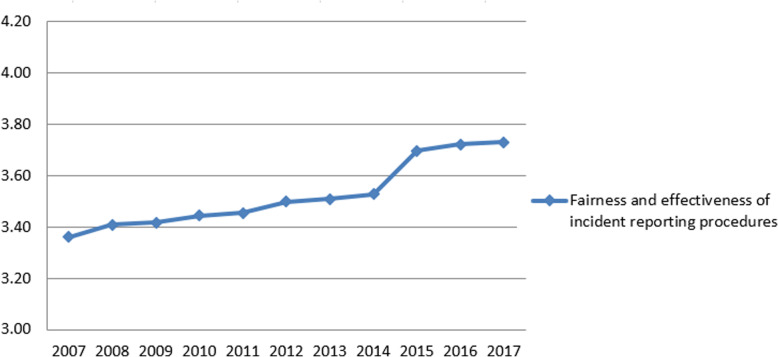
Annual average scores for NHS Staff Survey questions (scale score)

**Fig. 3:**
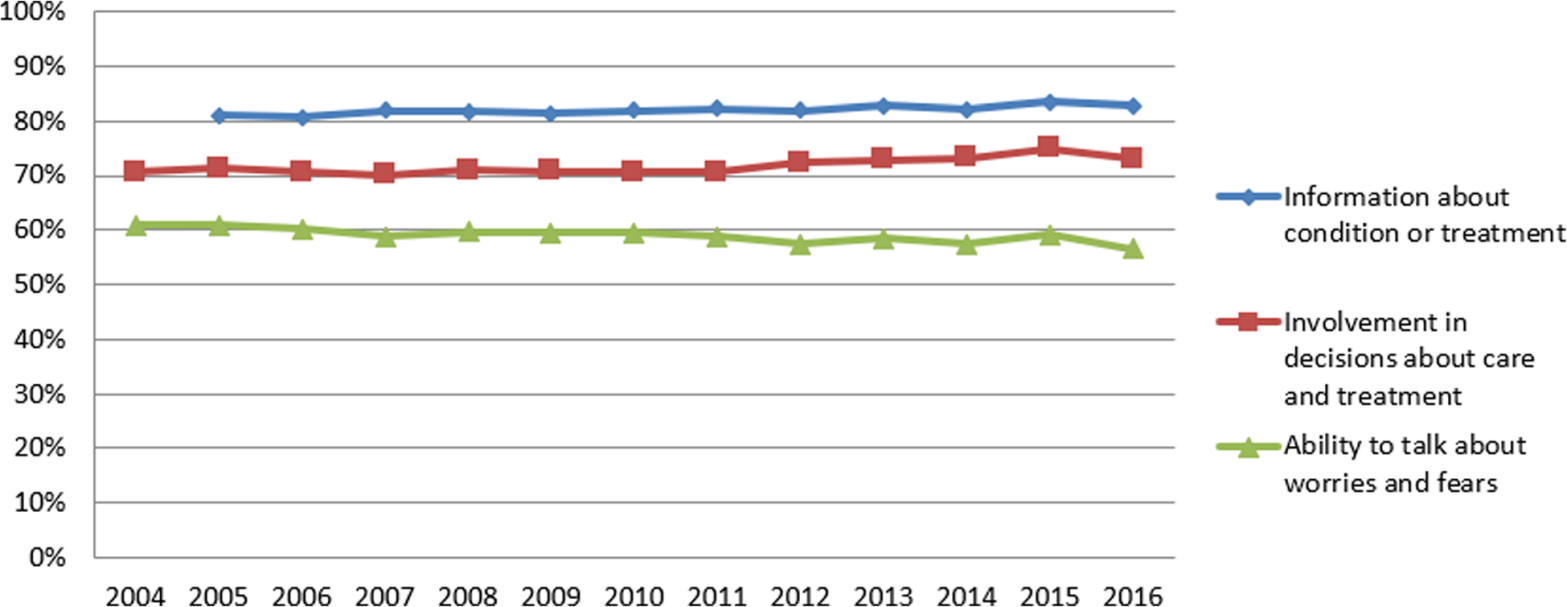
Annual average scores for NHS Acute Inpatient Survey questions

**Fig. 4:**
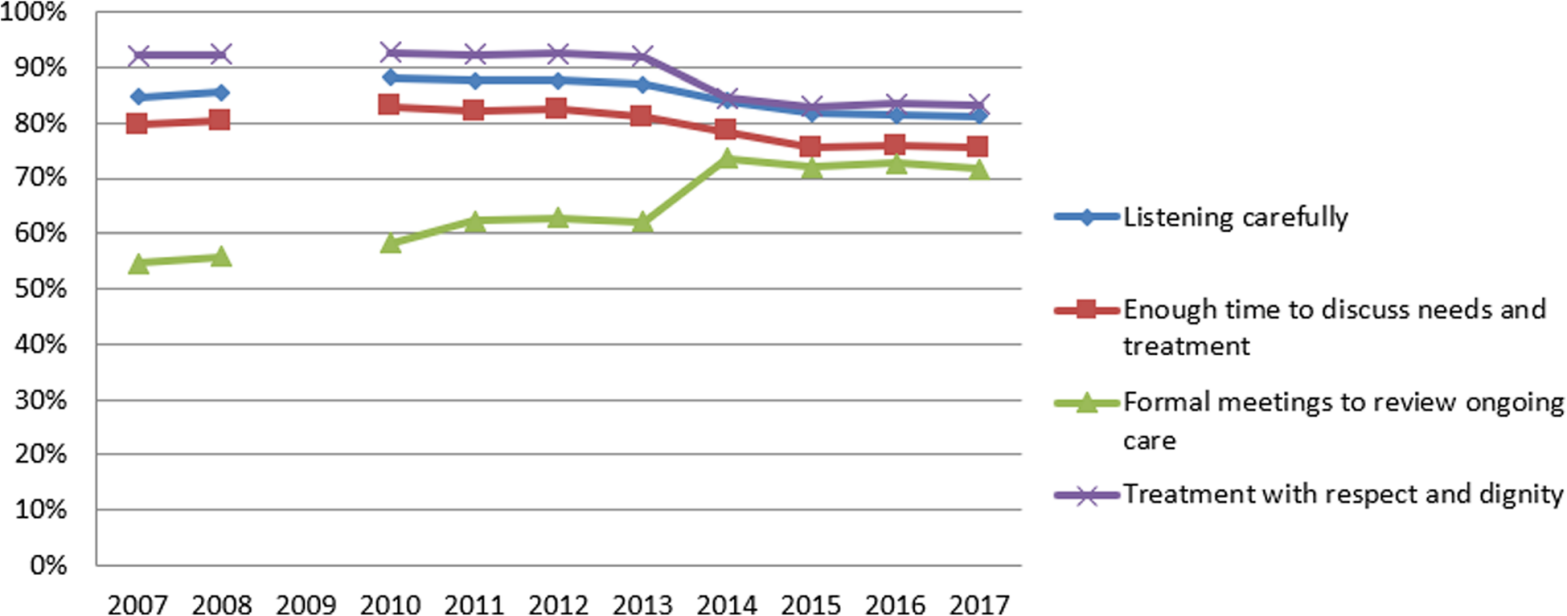
Annual average scores for NHS Community Mental Health Service User Survey questions

The correct figures have been included in this correction, and the original article has been corrected.
