# Supplementary material for: Openness in the NHS: a secondary longitudinal analysis of national staff and patient surveys
Source: BMC Health Serv Res. 2020 Sep 25;20:900. doi: 10.1186/s12913-020-05743-z (PMC7519560; doi:10.1186/s12913-020-05743-z)
Supplement: Supplementary file 1 — Additional file 1. [file 12913_2020_5743_MOESM1_ESM.docx]

**Supplementary Material 1: Descriptive statistics for all variables**

**1a. NHS Staff Survey variables**

| **Variable** | **Year** | **Mean (SD)** | **Median** | **Range** |
| --- | --- | --- | --- | --- |
| Good communication between managers and staff | 2008 | 26% (7%) | 26% | (4%, 57%) |
|  | 2009 | 27% (7%) | 26% | (6%, 62%) |
|  | 2010 | 28% (8%) | 27% | (5%, 59%) |
|  | 2011 | 27% (7%) | 27% | (6%, 52%) |
|  | 2012 | 28% (7%) | 28% | (8%, 52%) |
|  | 2013 | 30% (7%) | 30% | (9%, 47%) |
|  | 2014 | 30% (7%) | 30% | (8%, 51%) |
|  | 2015 | 31% (7%) | 31% | (12%, 51%) |
|  | 2016 | 33% (6%) | 33% | (11%, 48%) |
|  | 2017 | 33% (6%) | 33% | (14%, 48%) |
| Can contribute towards improvements | 2008 | 63% (8%) | 64% | (31%, 81%) |
|  | 2009 | 62% (8%) | 63% | (27%, 85%) |
|  | 2010 | 62% (8%) | 63% | (28%, 78%) |
|  | 2011 | 62% (7%) | 62% | (29%, 77%) |
|  | 2012 | 67% (7%) | 69% | (35%, 80%) |
|  | 2013 | 68% (7%) | 69% | (29%, 80%) |
|  | 2014 | 68% (7%) | 69% | (27%, 84%) |
|  | 2015 | 70% (6%) | 71% | (37%, 84%) |
|  | 2016 | 70% (6%) | 71% | (37%, 80%) |
|  | 2017 | 70% (6%) | 70% | (40%, 79%) |
| Fairness and effectiveness of incident reporting procedures | 2007 | 3.36 (0.11) | 3.36 | (2.81, 3.67) |
|  | 2008 | 3.40 (0.12) | 3.41 | (2.84, 3.68) |
|  | 2009 | 3.41 (0.12) | 3.43 | (2.89, 3.69) |
|  | 2010 | 3.44 (0.12) | 3.45 | (2.83, 3.75) |
|  | 2011 | 3.45 (0.11) | 3.46 | (2.90, 3.69) |
|  | 2012 | 3.50 (0.13) | 3.50 | (2.92, 3.77) |
|  | 2013 | 3.51 (0.12) | 3.52 | (2.94, 3.77) |
|  | 2014 | 3.52 (0.12) | 3.54 | (2.90, 3.77) |
|  | 2015 | 3.69 (0.13) | 3.71 | (3.07, 3.95) |
|  | 2016 | 3.72 (0.12) | 3.72 | (3.06, 4.00) |
|  | 2017 | 3.73 (0.12) | 3.73 | (3.17, 4.03) |

**1b. NHS Acute Inpatient Survey variables**

| **Variable** | **Year** | **Mean (SD)** | **Median** | **Range** |
| --- | --- | --- | --- | --- |
| Information about condition or treatment | 2005 | 81% (4%) | 80% | (73%, 93%) |
|  | 2006 | 81% (4%) | 80% | (70%, 97%) |
|  | 2007 | 82% (4%) | 82% | (69%, 96%) |
|  | 2008 | 82% (4%) | 82% | (57%, 95%) |
|  | 2009 | 81% (4%) | 81% | (72%, 97%) |
|  | 2010 | 82% (4%) | 82% | (71%, 95%) |
|  | 2011 | 82% (4%) | 82% | (70%, 97%) |
|  | 2012 | 82% (4%) | 82% | (71%, 96%) |
|  | 2013 | 83% (4%) | 83% | (74%, 95%) |
|  | 2014 | 82% (5%) | 82% | (56%, 97%) |
|  | 2015 | 84% (4%) | 83% | (73%, 97%) |
|  | 2016 | 83% (4%) | 82% | (74%, 96%) |
| Involvement in decisions about care and treatment | 2004 | 71% (5%) | 70% | (59%, 84%) |
|  | 2005 | 71% (5%) | 71% | (58%, 87%) |
|  | 2006 | 71% (5%) | 71% | (58%, 86%) |
|  | 2007 | 70% (5%) | 70% | (58%, 85%) |
|  | 2008 | 71% (5%) | 71% | (61%, 86%) |
|  | 2009 | 71% (5%) | 71% | (59%, 86%) |
|  | 2010 | 71% (5%) | 70% | (61%, 85%) |
|  | 2011 | 71% (5%) | 70% | (59%, 86%) |
|  | 2012 | 72% (5%) | 72% | (63%, 89%) |
|  | 2013 | 73% (5%) | 72% | (58%, 87%) |
|  | 2014 | 73% (5%) | 72% | (62%, 88%) |
|  | 2015 | 75% (5%) | 75% | (64%, 90%) |
|  | 2016 | 73% (5%) | 72% | (63%, 89%) |
| Ability to talk about worries and fears | 2004 | 61% (7%) | 61% | (43%, 81%) |
|  | 2005 | 61% (6%) | 61% | (42%, 81%) |
|  | 2006 | 60% (7%) | 60% | (44%, 81%) |
|  | 2007 | 59% (7%) | 58% | (41%, 80%) |
|  | 2008 | 60% (7%) | 60% | (41%, 80%) |
|  | 2009 | 59% (6%) | 59% | (47%, 80%) |
|  | 2010 | 60% (6%) | 60% | (40%, 81%) |
|  | 2011 | 59% (7%) | 58% | (42%, 79%) |
|  | 2012 | 58% (7%) | 58% | (41%, 78%) |
|  | 2013 | 58% (7%) | 58% | (39%, 81%) |
|  | 2014 | 57% (7%) | 56% | (44%, 78%) |
|  | 2015 | 59% (6%) | 58% | (44%, 78%) |
|  | 2016 | 57% (6%) | 55% | (45%, 77%) |

**1c. NHS Community Mental Health Service User Survey variables**

| **Variable** | **Year** | **Mean (SD)** | **Median** | **Range** |
| --- | --- | --- | --- | --- |
| Listening carefully | 2007 | 85% (2%) | 85% | (80%, 90%) |
|  | 2008 | 85% (3%) | 85% | (79%, 91%) |
|  | 2010 | 88% (2%) | 88% | (84%, 92%) |
|  | 2011 | 88% (2%) | 88% | (84%, 93%) |
|  | 2012 | 88% (2%) | 87% | (83%, 92%) |
|  | 2013 | 87% (2%) | 87% | (82%, 92%) |
|  | 2014 | 84% (3%) | 84% | (75%, 89%) |
|  | 2015 | 82% (3%) | 82% | (76%, 87%) |
|  | 2016 | 81% (3%) | 81% | (76%, 86%) |
|  | 2017 | 81% (3%) | 82% | (70%, 87%) |
| Enough time to discuss needs and treatment | 2007 | 80% (3%) | 80% | (72%, 85%) |
|  | 2008 | 80% (3%) | 80% | (74%, 88%) |
|  | 2010 | 83% (3%) | 83% | (76%, 89%) |
|  | 2011 | 82% (3%) | 82% | (74%, 89%) |
|  | 2012 | 82% (2%) | 82% | (77%, 87%) |
|  | 2013 | 81% (3%) | 81% | (74%, 87%) |
|  | 2014 | 78% (3%) | 78% | (72%, 84%) |
|  | 2015 | 75% (3%) | 75% | (68%, 80%) |
|  | 2016 | 76% (3%) | 75% | (67%, 84%) |
|  | 2017 | 75% (4%) | 75% | (59%, 82%) |
| Formal meetings to review ongoing care | 2007 | 55% (10%) | 53% | (37%, 75%) |
|  | 2008 | 56% (9%) | 56% | (26%, 78%) |
|  | 2010 | 58% (9%) | 59% | (42%, 81%) |
|  | 2011 | 62% (8%) | 63% | (35%, 84%) |
|  | 2012 | 63% (8%) | 64% | (48%, 77%) |
|  | 2013 | 62% (7%) | 62% | (50%, 80%) |
|  | 2014 | 74% (5%) | 73% | (63%, 82%) |
|  | 2015 | 72% (5%) | 73% | (58%, 86%) |
|  | 2016 | 73% (5%) | 73% | (59%, 83%) |
|  | 2017 | 72% (5%) | 72% | (59%, 83%) |
| Treatment with respect and dignity | 2007 | 92% (2%) | 93% | (88%, 96%) |
|  | 2008 | 92% (2%) | 92% | (87%, 97%) |
|  | 2010 | 93% (2%) | 93% | (87%, 97%) |
|  | 2011 | 92% (2%) | 92% | (87%, 96%) |
|  | 2012 | 93% (2%) | 92% | (88%, 96%) |
|  | 2013 | 92% (2%) | 92% | (87%, 95%) |
|  | 2014 | 84% (3%) | 84% | (79%, 90%) |
|  | 2015 | 83% (3%) | 83% | (76%, 89%) |
|  | 2016 | 84% (3%) | 83% | (77%, 90%) |
|  | 2017 | 83% (4%) | 84% | (71%, 88%) |

**Supplementary Material 2: Fit statistics for competing longitudinal models**

**1a. NHS Staff Survey variables**

| **Variable** | **Model** | **CFI** | **RMSEA** |
| --- | --- | --- | --- |
| Good communication between managers and staff | 1 | 0.940 | 0.106 |
|  | 2 | 0.961 | 0.087 |
|  | 3 | 0.974 | 0.073 |
|  | 4 | 0.965 | 0.063 |
|  | 5 | 0.969 | 0.061 |
| Can contribute towards improvements | 1 | 0.855 | 0.194 |
|  | 2 | 0.858 | 0.195 |
|  | 3 | 0.858 | 0.201 |
|  | 4 | 0.869 | 0.149 |
|  | 5 | 0.875 | 0.147 |
| Fairness and effectiveness of incident reporting procedures | 1 | 0.794 | 0.225 |
|  | 2 | 0.811 | 0.219 |
|  | 3 | 0.831 | 0.213 |
|  | 4 | 0.831 | 0.161 |
|  | 5 | 0.832 | 0.161 |

Notes:

- Model 1 is a piecewise growth curve model (PGCM) with fixed slopes either side of 2013 (no control variables included)
- Model 2 is a PGCM with fixed slopes before 2013, and random slopes thereafter (no control variables included)
- Model 3 is a PGCM with random slopes either side of 2013, but no control variables included
- Model 4 is a PGCM with random slopes either side of 2013 and control variables included (the PGCM reported in the main article)
- Model 5 adds in a random step change from 2013 to 2014 so it becomes an interrupted time series latent growth model (ILGM) – this is the ILGM reported in the main article

**1b. NHS Acute Inpatient Survey variables**

| **Variable** | **Model** | **CFI** | **RMSEA** |
| --- | --- | --- | --- |
| Information about condition or treatment | 1 | 0.879 | 0.089 |
|  | 2 | 0.879 | 0.090 |
|  | 3 | 0.884 | 0.090 |
|  | 4 | 0.865 | 0.087 |
|  | 5 | 0.885 | 0.080 |
| Involvement in decisions about care and treatment | 1 | 0.907 | 0.127 |
|  | 2 | 0.907 | 0.128 |
|  | 3 | 0.910 | 0.128 |
|  | 4 | 0.901 | 0.114 |
|  | 5 | 0.900 | 0.114 |
| Ability to talk about worries and fears | 1 | 0.950 | 0.091 |
|  | 2 | 0.949 | 0.093 |
|  | 3 | 0.960 | 0.084 |
|  | 4 | 0.952 | 0.077 |
|  | 5 | 0.952 | 0.078 |

Notes:

- Model 1 is a piecewise growth curve model (PGCM) with fixed slopes either side of 2013 (no control variables included)
- Model 2 is a PGCM with fixed slopes before 2013, and random slopes thereafter (no control variables included)
- Model 3 is a PGCM with random slopes either side of 2013, but no control variables included
- Model 4 is a PGCM with random slopes either side of 2013 and control variables included (the PGCM reported in the main article)
- Model 5 adds in a random step change from 2013 to 2014 so it becomes an interrupted time series latent growth model (ILGM) – this is the ILGM reported in the main article

**1c. NHS Community Mental Health Service User Survey variables**

| **Variable** | **Model** | **CFI** | **RMSEA** |
| --- | --- | --- | --- |
| Listening carefully | 1 | 0.224 | 0.186 |
|  | 2 | 0.236 | 0.190 |
|  | 3 | 0.300 | 0.189 |
|  | 4 | 0.207 | 0.175 |
|  | 5 | 0.226 | 0.174 |
| Enough time to discuss needs and treatment | 1 | 0.292 | 0.181 |
|  | 2 | 0.280 | 0.187 |
|  | 3 | 0.390 | 0.179 |
|  | 4 | 0.363 | 0.154 |
|  | 5 | 0.360 | 0.155 |
| Formal meetings to review ongoing care | 1 | 0.079 | 0.236 |
|  | 2 | 0.170 | 0.226 |
|  | 3 | 0.189 | 0.230 |
|  | 4 | 0.033 | 0.198 |
|  | 5 | 0.098 | 0.192 |
| Treatment with respect and dignity | 1 | 0.000 | 0.278 |
|  | 2 | 0.000 | 0.277 |
|  | 3 | 0.000 | 0.288 |
|  | 4 | 0.000 | 0.243 |
|  | 5 | 0.118 | 0.206 |

Notes:

- Model 1 is a piecewise growth curve model (PGCM) with fixed slopes either side of 2013 (no control variables included)
- Model 2 is a PGCM with fixed slopes before 2013, and random slopes thereafter (no control variables included)
- Model 3 is a PGCM with random slopes either side of 2013, but no control variables included
- Model 4 is a PGCM with random slopes either side of 2013 and control variables included (the PGCM reported in the main article)
- Model 5 adds in a random step change from 2013 to 2014 so it becomes an interrupted time series latent growth model (ILGM) – this is the ILGM reported in the main article
